# Should implementation intentions interventions be implemented in obesity prevention: the impact of if-then plans on daily physical activity in Dutch adults

**DOI:** 10.1186/1479-5868-6-11

**Published:** 2009-03-06

**Authors:** Emely De Vet, Anke Oenema, Paschal Sheeran, Johannes Brug

**Affiliations:** 1 Department of Health Sciences and EMGO institute for Health and Care Research, Faculty of Earth and Life Sciences, VU University Amsterdam, De Boelelaan 1085, 1081 HV Amsterdam, the Netherlands; 2 Department of Public Health, Erasmus MC University Medical Center, PO Box 2040, 3000 CA Rotterdam, the Netherlands; 3 Department of Psychology, University of Sheffield, Sheffield, S10 2TN, UK; 4 EMGO institute for Health and Care Research, VU University Medical Center, Van der Boechorststraat 7, 1081 BT Amsterdam, the Netherlands

## Abstract

**Background:**

Forming implementation intentions (specifying when, where and how to act) has been proposed as a potentially effective and inexpensive intervention, but has mainly been studied in controlled settings for straightforward behaviors.

**Purpose:**

To examine if forming implementation intentions (II) could be used in large-scale, population-based interventions that aim to promote more complex and clinically relevant behavior change, we tested the impact of different II on increasing daily physical activity (PA) aimed at weight maintenance among 709 Dutch adults.

**Methods:**

At T0, participants were randomly allocated to a control group or to form II for 1) a prescribed action (walking), 2) self-selected activities, 3) self-selected activities and repeat making these II two times. All participants were asked to increase PA by at least two hours a week (15–20 minutes per day). Post-tests took place two weeks (response 85%), three months (response 78%) and six months (response 79%) post-intervention.

**Results:**

No main effects of II formation on BMI or physical activity were found. Intention to increase physical activity moderated the effects of repeated II, but not of the other II conditions. Forming repeated II had a positive effect on total PA and number of active days for respondents with strong intentions.

**Conclusion:**

Implementation intention interventions may not yet be ready for implementation on its own for large-scale obesity prevention in the general public. Future research should test strategies for optimal II formation in both initiating and maintaining behavioral change.

**Trial registration:**

ISRCTN81041724

## Background

Helping people to avoid becoming overweight (BMI ≥ 25 kg/m^2^) and obese (BMI ≥ 30 kg/m^2^) is an important public health priority, since the prevalence of excess body weight is high [1], and obesity is clearly associated with chronic diseases, such as cardiovascular diseases, type 2 diabetes, and certain types of cancers [2-4]. Excess weight originates from a long-term positive energy balance in which energy intake (from diet) exceeds energy expenditure (mainly physical activity). It has been estimated that adults gain on average 1.8 to 2.0 pounds per year, which is caused by an energy imbalance of only 100 kcal per day [5]. If this energy imbalance of 100 kcal per day could be restored, weight gain could be prevented in approximately 90% of the population. The energy balance could be restored by, for instance, increasing life-style physical activity (e.g. walking) by only 15 to 20 minutes per day or approximately two hours per week [5].

Many people express positive intentions to increase their physical activity levels. However, many people do not act upon their positive intentions to change their lifestyles [6]. Forming implementation intentions (II) has been proposed as a potentially effective and inexpensive intervention, particularly suited to help people to act upon their positive intentions [7,8]. Implementation intentions are if-then plans specifying when, where and how one will act in order to achieve a goal ("If I encounter situation X, then I will perform behavior Y"; e.g. "If I arrive at work in the morning, then I will take the stairs instead of the elevator to my workplace"). By forming II individuals commit themselves to acting as soon as the specified situation is encountered. Usually II interventions consist of writing down when, where and how one will act to achieve an intended goal. Meta-analyses showed that II interventions may be a powerful tool in changing a range of health behaviors [6,9,10]. A large meta-analysis by Gollwitzer and Sheeran of *k *= 94 independent tests among a total of 8461 participants showed that II had a positive effect of medium-to-large magnitude (*d *= .65) on goal attainment [9]. In the present study, we tested the impact of implementation intention interventions aimed at increasing physical activity by at least two hours per week in order to achieve weight maintenance or modest weight loss. If effective, this strategy could easily be implemented in large-scale population-based obesity-preventive interventions.

Although only a few studies examined the effect of II on physical activity, most of these studies revealed promising results of II [e.g. [11-14]]. However, several issues remain to be addressed before implementation intentions for physical activity can be applied in obesity prevention in the general public. First, the conducted trials targeted exercise or vigorous physical activity (i.e. specific categories of total physical activity) [e.g. [13]]. Weight maintenance, however, does not necessarily imply an increase in exercise, but can be achieved by an increase in all sorts of daily activities, including low-intensity activities [15,16]. In fact, evidence suggests that increasing high-intensity exercise does not result in higher total energy expenditure, since such high-intensity exercise is often compensated by extra rest [17]. Second, past studies tested II for specific and straightforward actions unlikely to result in clinically relevant changes in energy balance, such as one extra exercise session in the next week [12] or watching an exercise video once [18]. Implementation intentions were thus formed for a very specific target assigned by the researchers. Total daily physical activity is a more complex category of behaviors, and includes a variety of actions (e.g. going to work by bike or foot, household work, exercise, walking, gardening). When forming II to increase total physical activity, people first have to decide what activity they chose to perform. A small meta-analysis suggested that implementation intentions were effective in pursuing self-generated goals, but that effect sizes of II were generally smaller for self-generated goals than for assigned goals [19]. However, to date no studies directly compared the effect of II for an assigned versus a self-selected goal. We therefore tested two types of implementation intentions aimed at increasing physical activity, i.e., II for an assigned activity (walking) and II for a self-selected activity. We hypothesized that II for the assigned activity would be more effective than II for the self-selected activity. Third, people need to maintain physical activity for a longer period of time in order to prevent weight gain. Past research, however, has indicated that forming implementation intentions is more effective in studies with short-term follow-ups than for long-term goals [19]. II are cognitive strategies that might be forgotten or become irrelevant over time [19]. It might also be that anticipated situations and barriers may differ over time and become different from the situations and barriers anticipated in the original II. To date only effects of forming II at one point in time have been tested. It may well be that for longer-term goals, II should be formed more than once to establish clinically relevant changes in physical activity. In this study the effects of single and repeated formation of II was tested. We hypothesized that repeated formation of II would be more effective than single II formation for increasing physical activity. Fourth, previous trials were conducted in rather controlled settings (e.g. one-to-one sessions with consultants) and in selected groups (e.g. students or patients) [11,20,21], but not in the open, general adult population. For example, patients in rehabilitation from myocardial infarction formed implementation intentions for increasing moderate-intensity physical activity. An interviewer asked to provide more details about their plans (e.g. type of exercises, exact time, and exact circumstances) and gave supportive feedback about the plans [11]. Although this procedure may be very useful in clinical settings and in high-risk approaches, it appears unfeasible for large-scale population-based interventions. We, therefore, examined II in a setting that could resemble that of a large-scale intervention. Finally, many past experimental studies encouraged respondents in an implementation intention condition to change behavior and to plan this change as precisely as possible, whereas respondents in the control condition only completed questionnaires without general encouragement to make behavior changes [e.g. [22]]. Such a design does not actually allow for assessing the unique effect of II, but assesses the effect of encouragement to change plus an II manipulation. To be able to assess the unique effect of II, in the present study also the control group was encouraged to increase their total physical activity.

In a randomized controlled trial, we tested whether forming implementation intentions to increase total physical activity by two hours per week had an impact on BMI and increased physical activity at two weeks, three and six months follow-up. Because II theory posits that forming II is a useful strategy for translating strong intentions into actual behavior change [7,8], we hypothesized that II will be effective for individuals with strong intentions, but not for individuals with weak intentions to increase their PA by two hours per week.

More specifically, we hypothesized that:

1) Forming II leads to a greater decrease in BMI, and greater increases in physical activity (PA) than not forming II.

2) Forming II for an assigned activity (i.e. walking) is more effective than forming II for self-selected activities.

3) Repetition of forming II to increase PA is more effective than forming II once.

4) The effects of II will be moderated by the intention to increase physical activity.

## Method

### Participants, procedures, and design

Eligible participants, between 18 and 65 years of age, could indicate their willingness to participate in the entire study by responding to invitation announcements in newsletters and at worksites' intranet and mail. Recruitment took place in 2003 and 2004. After responding to the invitation, participants were sent the first questionnaire either to their home or work address. Of the participants 40.5% were recruited in response to newsletters, and 59.5% in response to worksites' intranet or mail. In order to detect a 2 kg weight difference (power 80%, p < .05), 100 participants are needed in each condition. To allow for drop-out extra participants were recruited. In total, 709 respondents completed the pretest questionnaire and signed the informed consent form that was printed on a separable sheet in the questionnaire. Questionnaires and informed consent forms could be returned by prepaid envelope. Four types of questionnaires were distributed. All questionnaires were identical except for the final part where each questionnaire corresponded to one experimental condition: 1) make II for an assigned activity (i.e. walking), 2) make II for self-selected activities at one point in time, 3) repeat making II for self-selected activities two times (two weeks and after three months), and 4) control group. The four types of questionnaires were stacked in a computer-determined random order, and were disseminated unblinded to participants in that order. Twenty-five gift vouchers of 20 euros were raffled amongst participants who completed the entire study.

Post-tests took place two weeks (T1: n = 600, response rate 85%), three months (T2: n = 555, response rate 78%) and six months post-intervention (T3: n = 562, response rate 79%). In total, 72% of the respondents (n = 511) completed all four questionnaires.

An institutional ethical review board approved the study protocol.

### Study conditions

At pre-test, all questionnaires started by explaining the association between total physical activity, health, and excess body weight. It was mentioned that experts had calculated that if all Dutch people would increase their physical activity by two hours per week, the number of overweight people would decrease dramatically. All participants, including the control group (n = 206; 29%), were asked to increase their physical activity level by at least two hours per week or 15 to 20 minutes per day. In addition, respondents in one of the three II groups were told that to really increase their physical activity/walking activity by an extra two hours per week, it is helpful to explicitly decide on the activities one would engage in and on what day(s), when, where and for how long they would do the activities and they were asked to make such a plan. Respondents were presented the following *"Please decide now, and write down in the scheme below: 1) what activity you will do, 2) on what day or days you plan to do the activity, 3) when you will do the activity, 4) where you will do the activity, and 5) how long you will perform the activity at your selected moments"*.

In the implementation intention for an assigned activity (n = 161, 23%) condition, participants were told that walking would be a good activity to increase their PA, that you could walk at any place and at any time, and that special sporting equipment is not necessary. They were told that to really increase their physical activity by walking an extra two hours, it is helpful to explicitly decide on what day(s), when, where and how long they would walk. All components were explained in detail. For example, it was stated that *when *should not only concern a particular time of the day but rather a moment on the day, such as after getting up, before or after work, before breakfast, during lunch break. *Where *involved specifying the place of the activity, such as in the park, at a sports field, in the neighborhood. *How long *indicated the amount of time they would spend walking at each of the specified moments. This latter component was included so participants could calculate how to achieve the additional two hours of physical activity.

This procedure was also employed in the implementation intention for self-selected activities (n = 172, 24%) and in the repeated implementation intentions for self-selected activities (n = 170, 24%) study conditions. Participants in these study conditions were first asked to select activities (up to three) they would engage in to increase physical activity by two hours per week. Next, they were asked to make II for these self-selected activities. Participants in the repeated implementation intentions for self-selected activities study condition were asked to repeat making II again at the end of the T1 and T2 follow-up questionnaires, following the same instructions. All participants were asked to continue to increase their PA levels.

In all three implementation intention groups, participants could write down the various components of the II on a pre-structured form with blank lines for writing down the II components. Respondents were provided the opportunity to specify up to three II. Respondents were asked to reread the implementation intentions before sending back the questionnaire. The II instructions were comparable to earlier studies [e.g. [23]], and were adapted to the target of daily physical activity whenever appropriate.

### Measures

Self-reported weight was measured at every assessment by asking people to indicate their weight in kilograms. Participants reported height in centimeters at baseline. By dividing weight by the square length in meters, the *body mass index *(BMI) was calculated for all measurements.

*Physical activity *was measured at all four assessments using the Dutch Short Questionnaire to Assess Health enhancing physical activity (in short; SQUASH) [24]. The questionnaire includes questions about frequency, duration, and intensity of activities in four domains of physical activity, i.e. transportation (walking and bicycling to work), activities at work, household activities, leisure-time activities (gardening, walking, bicycling, doing odd jobs, fitness, running, swimming, and other sports). Respondents are asked to report 1) on how many days in the past week they performed the activity, 2) the average time spent in the specific activity on such a day, and 3) the intensity by which the activity was performed (light, moderate, heavy). The SQUASH has been validated against a CSA activity monitor (*r *= .45) [24]. Physical activity in the last week was measured. At all four assessments, we calculated total physical activity, moderate-intensity physical activity, and walking in minutes per week from the SQUASH.

*Number of active days *was assessed by asking respondents to indicate the average number of days per week they engage in at least 30 minutes of moderate-intensity physical activity per day.

*Intention to increase physical activity *was measured at T0 using two items: "Do you intend to increase your physical activity with two hours a week" and "Are you willing to increase your physical activity with two hours a week" (Definitely not [-2] – Definitely yes [2]). Next to intention to increase physical activity, also intention to walk two hours a week more were assessed with these same items. Cronbach's alphas were .88 and .87 for intention to increase physical activity and walking, respectively. Mean scores were computed.

*Demographic information *such as age, sex, ethnicity, level of education were assessed only at T0.

### Statistical analyses

Descriptive statistics were used to describe the study population at baseline with respect to demographic characteristics, BMI, total physical activity, moderate-intensity physical activity, walking, and number of active days. Cook's distances were calculated and values larger than 1.00 would indicate outliers [25]. Next, to test whether randomization was successful, analysis of variance and Chi-square tests were used to detect differences between the experimental conditions in age, sex, level of education and ethnicity, and baseline BMI and physical activity. These same variables, and additionally intervention condition were further entered as independent variables in logistic regression analyses with dropout (1 = did not complete all four assessments, and 0 = did complete all four assessments) as the dependent variable, in order to detect selective dropout.

The first hypothesis was tested by means of repeated measures ANOVA. In these analyses we tested the effects of II on BMI, total physical activity, moderate-intensity physical activity, walking and number of active days. For each of these outcome measures, time, condition and the time × condition interaction were entered as the independent variables. If one of the independent variables revealed a (marginally [*p *< .10]) significant result, this result was decomposed using simple and repeated contrast analyses to gain more specific insight into differences between conditions, time intervals and on what time interval the interventions had any effects. With these contrast analyses, hypothesis two and three could be tested.

To test hypothesis four, multiple linear regression analyses were conducted. In these regression analyses, total physical activity, moderate-intensity physical activity, walking, and number of active days at T1 were the dependent variables, respectively. Baseline activity levels, the assumed standardized moderator (intention), intervention condition, and the multiplicative standardized moderator by intervention condition term were entered as the independent variables. If an interaction term had a *p*-value < .10, this interaction was decomposed as specified by Aiken and West [26]. Simple slopes for implementation intentions were computed at two levels of the moderator: e.g. low intention (M-1SD) and high intention (M + 1SD). It should be noted that the intention measure corresponding to the outcome measure was used in the analysis. That is, for the moderation analyses with total PA as the outcome measure, intention to increase PA was entered as the moderator. Intention to walk two hours more per week was entered in the regression, when walking was the outcome measure.

All tests were two-tailed and alpha levels were set at .05, unless indicated otherwise. All analyses were conducted using SPSS 11.0.

## Results

### Participants

The study sample consisted of 709 adults, of which 67% were women and 33% men (n = 477 women, n = 231 men, n = 1 missing). Mean age was 45.90 (SD = 10.34). Most respondents were of Dutch ethnicity (90%). Of the respondents, 15% had a low level of education (completed no education, primary school, secondary school, or lowest level of high school or lower vocational training), 19% had a medium level of education (completed intermediate or high level high school, or medium level vocational training), and 66% had a high level of education (completed higher vocational training, college or university training had a high level of education).

Mean BMI was 24.25 (SD = 3.69), and 37% of the respondents were overweight (BMI = 25). According to the SQUASH, respondents' total physical activity averaged 2766 (SD = 1149) minutes per week, which is somewhat lower compared to the 3045 (931) minutes per week found in the validation study by Wendel-Vos and coworkers [24]. Of the reported time in our study, 48% concerned activities at work or school, and 32% concerned household activities. Leisure time, sporting and commuting activities reflected 19%, 5% and 3% of the reported time, respectively. Overall, 18% of the reported time concerned moderate-intensity activities. In total, 56.9% of sample reported to be active for minimal 30 minutes per day on at least five days in the week. Table 1 provides a comparison of sample characteristics with the general Dutch population. Cook's distances for the various outcome measures varied from .00 to .47, so no indications for outliers were found. No differences between the four conditions were found in sex, age, ethnicity, level of education, BMI, or PA, indicating that randomization was successful. Further, no differences were found between completers and non-completers with respect to sex, ethnicity, level of education, BMI, PA, but older participants were more likely to complete the study than younger participants (Completers mean age was 47 years, Non-completers mean age was 42 years; OR = 0.96, *p *< .001).

**Table 1:** Characteristics of the Study Sample compared to the Dutch Population

		Study sample	Dutch population
Sex	Males	33%	49%
	Females	67%	51%
Level of education	Low	15%	35%
	Medium	19%	41%
	High	66%	25%
Ethnicity	Dutch origin	90%	80%
	Foreign origin	10%	20%
Prevalence of overweight		37%	45%
Prevalence of Dutch norm of physical activity		57%	56%

### Effects of II on BMI and Physical Activity measures

Of the respondents who were asked to form II, 89%, 89% and 86% did so at T0, T1, and T2, respectively. Table 2 presents descriptive data on BMI and physical activity-related outcome measures for each of the intervention groups and assessments. Repeated measures ANOVA showed no effects of condition or time × condition effects on BMI, total PA, moderate-intensity PA, walking, and number of active days (Table 3). For moderate-intensity PA, walking, and number of active days, but not for BMI and total PA, (marginally) significant time effects were found (Table 2). For the PA-related measures, activity increased between T0 and T1, and was still higher at T2 and T3 than at T0 (Table 3).

**Table 2:** Mean (M) and Standard deviation (SD) of BMI and physical activity measures by intervention group and assessment

	**Control group**		**II for walking ** **group**		**II self-selected ** **activity group**		**II repeated ** **group**	
**T0**	**M**	**SD**	**M**	**SD**	**M**	**SD**	**M**	**SD**

BMI (kg/m^2^)	24.42	3.88	24.28	3.53	24.35	3.44	23.93	3.86

Total activity (minutes/week)	2789	1163	2748	1076	2777	1118	2745	1237

Walking (minutes/week)	124	161	146	232	140	208	127	226

Moderate-intensity PA (minutes/week)	519	657	500	490	441	544	435	475

Number of active days (days/week)	4.58	2.14	4.81	2.23	4.75	2.16	4.65	2.09

**T1**								

BMI	24.40	3.83	24.39	3.68	24.20	3.21	23.88	3.90

Total activity	2654	1143	2669	1132	2710	1272	2724	1435

Walking	154	224	164	179	194	265	129	150

Moderate-intensity PA	539	585	515	561	453	481	450	456

Number of active days	5.02	2.06	5.10	2.03	4.81	2.34	5.01	2.14

**T2**								

BMI	24.70	3.82	24.29	3.38	24.18	3.24	24.07	4.10

Total activity	2700	1320	2791	1494	2742	1207	2605	1198

Walking	154	213	172	217	153	210	156	219

Moderate-intensity PA	535	539	560	577	489	505	489	546

Number of active days	5.12	2.11	4.96	2.23	5.27	2.11	4.95	2.11

**T3**								

BMI	24.47	3.76	24.04	3.24	24.33	3.32	24.27	4.07

Total activity	2870	1482	2745	1240	2707	1240	2729	1254

Walking	183	391	172	236	189	538	131	169

Moderate-intensity PA	562	650	562	516	465	458	518	542

Number of active days	5.10	2.05	5.07	2.14	5.06	2.10	5.17	1.96

**Table 3:** Effects of interventions on BMI and physical activity outcome measures

		**Time ^a^**	**Condition**	**Time × condition**	**Contrast analyses**
**Outcome measures**	**n **^b^	**F-value**	**F-value**	**F-value**	

BMI	475	0.33	0.64	0.84	NA
Total physical activity	495	0.86	0.26	0.39	NA
Moderate-intensity PA	499	2.53^‡^	1.71	0.39	T0 < T1, T2 T3
Walking	504	2.74^‡^	1.01	0.64	T0 < T1, T2 T3
Number of active days	369	15.32***	0.73	0.68	T0 < T1, T2 T3

Note ^‡ ^*p *< .10, * *p *< .05, ** *p *< .01, *** *p *< .001. Data collection started in 2003 in the Netherlands

^a ^Repeated measures ANOVA

^b ^Numbers of respondents (n) refer to all respondents included in the analyses.

### Are II effects moderated by intention?

Intention to increase physical activity moderated the effects of repeated II, but not of the other II conditions, on total PA and number of active days at T1 (Table 4). For low levels of intention, forming repeated II had no effect on total PA (B = -158.49, *p *= .39) or number of active days (B = -0.54, *p *= .10). Forming repeated II had a (marginally) significant positive effect on total PA (B = 339.90, *p *= .06) and number of active days (B = 0.69, *p *= .04) for high levels of intention."

**Table 4:** Moderated regression of outcomes at T1, on intention, condition and interactions

	**Total PA**	**Moderate-** **intensity PA**	**Walking**	**Number of ** **active days**
**Variable entered**	**Bèta**	**Bèta**	**Bèta**	**Bèta**

Intention	-.01	-.08	-.04	-.11
II repeated	.04	-.02	-.06	.02
II repeated × intention	.13 ‡	.06	.04	.19**
R^2^	.21	.48	.25	.23
F	20.11***	72.17***	26.16***	19.71***

*Note*. All analyses were corrected for the baseline value of the outcome measure.

^‡ ^*p *< .10, * *p *< .05, ** *p *< .01, *** *p *< .001

## Discussion

The present randomized controlled trial tested the impact of various II on clinically relevant changes in complex health behavior, i.e., daily PA, among a sample of Dutch adults. In general, we did not find effects of II formation on any of the outcome measures. Physical activity increased equally among those who did and who did not form II. Further, forming II for a prescribed specific action (i.e. walking) or making repeated II did not result in greater increases in PA, compared to either forming a single II for self-selected activities or not forming II at all.

Several explanations can be offered for the lack of II effects in our study. First, because we aimed to evaluate the potential of II for application in the general public, participants formed II autonomously. In various earlier II studies on exercise, II formation was facilitated by personal counseling – a setting in which the formulation of II is controlled and guided, and allows immediate checking of the quality of the II. It may be that II are not specific enough or that the quality of plans is less than optimal when individuals form II on their own. If individuals specify a situation in their implementation intention that is rather ambiguous or is not encountered frequently (e.g. I will go running when the weather is nice), the effects of a specific plan on behavior may be diminished. However, for population-based application of II only self-formed II may be feasible. This study indicates that the efficacy of formation of II for complex behaviors outside controlled settings is not yet established.

Second, as outlined in the introduction, the purpose of the present study was to assess the unique effect of II. Therefore, also the control group was encouraged to increase their total physical activity by at least two hours per week. Our findings showed that physical activity increased in all conditions during the six-month study period. It might be that general encouragement was sufficient to ensure increased physical activity for many participants, and that forming II conferred no additional benefit or that participants in the control group formed spontaneous II to increase their PA [27]. It is worthwhile noting that a previous study with a design similar to our study also did not find effects of II on fruit and vegetable intake, even though there was an increase in fruit and vegetable intake over time [28].

Third, participants formed II that were geared towards helping participants to initiate physical activity – they specified when, where, and how they would increase their physical activity. However, people may start with extra physical activity, but fail to maintain extra physical activity due to various kinds of barriers, temptations or distractions (e.g. a lack of time, bad weather, being tired). The implication is that participants in the present study could have benefited not only from forming II to get started, but also by forming II to overcome barriers or other unwanted influences. For instance, participants could have specified precisely how they would react to particular temptations or distractions (e.g., "And if I feel tired when I come home, then I will ignore it and go for my walk"). To overcome or prevent self-regulatory problems, individuals need to get acquainted with how to make plans and how to adapt plans to regulate their daily behaviors. The single act of completing an implementation intention sheet and mentally representing the formed II may be distinct from the use of a planning strategy in regulating daily behaviors in everyday life [11]. An RCT evaluated the effects of an II intervention on physical activity among myocardial infarction patients. The patients in the II group were trained how to form and adapt plans. After the II intervention, the patients more often used planning in their daily lives, which in turn increased physical activity six months later [11].

Fourth, the moderation analyses suggested that forming repeated implementation intentions may have some effects on physical activity at T1, but only for those with a positive intention to increase physical activity. These findings are consistent with previous evidence indicating that strong II effects only emerge when goal intentions are activated and strong [9]. Remarkably, the effects of forming single II at T1 were not moderated by intentions, while at T1 respondents in both repeated and single II groups had formed II only once. Overall, the moderation analyses suggest that implementation intention interventions may need to be supported by motivational interventions in order to have an impact on physical activity and BMI. Future research should aim to test whether such a combined motivation plus II intervention could promote weight control.

### Study limitations and strengths

The present study has certain limitations. First, the study sample has more females, more respondents of Dutch origin, and more respondents with a high level of education as compared to the general Dutch population. Although the prevalence of overweight and physical inactivity in the sample did not deviate substantially from the Dutch population, the external validity of the results might be limited. Second, although the SQUASH has been validated against a CSA activity monitor [24], validity does not guarantee that the SQUASH also has sufficient sensitivity. However, the present study and one previously published intervention study employing the SQUASH [29], found changes in physical activity over time, which is an indication of sufficient sensitivity. Nevertheless, the use of more objective PA measures (e.g. accelerometer data) would have improved internal validity. However, such objective PA assessments are difficult to obtain in larger scale population-based studies and may reduce external validity [30].

An important strength is that the study was designed to detect clinically relevant effects in total PA behavior, energy expenditure and BMI. Past studies examined II for specific actions unlikely to result in actual changes in energy balance, such as one extra exercise session in the next week [12] or watching an exercise video once [18]. A simple strategy as II might be effective for straightforward actions that do not require much effort from an individual, but may not be powerful enough to produce clinically relevant changes in a complex behavior.

## Conclusion

The present trial showed no effect of II formation on total physical activity or BMI. This might suggest that standard II interventions that ask people to specify when, where, and how they will be more physically active may not be a panacea for clinically relevant increases in PA among members of the public such as those sampled in the present study. However, the lack of effects of II may also be based on several other reasons, such as the II format, the selectivity of the sample or the validity and reliability of the outcome measures used. Therefore, it cannot be concluded that II interventions are ineffective in increasing physical activity. Despite the lack of effects, our study may still help to refine future research on how II can be effectively implemented in public health interventions. For example by testing different strategies for II formation (e.g. training in using planning strategies) or testing different types of II (e.g. II to prevent relapse). In conclusion, implementation intention interventions may not yet be ready to be implemented in obesity prevention. There needs to be careful consideration of the motivational challenges and self-regulatory problems that participants face before II interventions can be effectively implemented in large-scale weight-control programs that aims making changes of clinically-relevant magnitude.

## Abbreviations

II: implementation intentions; PA: physical activity

## Competing interests

The authors declare that they have no competing interests.

## Authors' contributions

EV performed the statistical analysis and drafted the manuscript. AO, PS and JB participated in the design of the study and helped to draft the manuscript. All authors read and approved the final manuscript.
